# Case Report: A child with NFKB1 haploinsufficiency explaining the linkage between immunodeficiency and short stature

**DOI:** 10.3389/fimmu.2023.1224603

**Published:** 2023-08-03

**Authors:** S. Ricci, S. Abu-Rumeileh, N. Campagna, F. Barbati, S. Stagi, C. Canessa, L. Lodi, B. Palterer, L. Maggi, A. Matucci, A. Vultaggio, F. Annunziato, C. Azzari

**Affiliations:** ^1^ Department of Health Sciences, University of Florence, Florence, Italy; ^2^ Immunology Division, Section of Pediatrics, Meyer Children’s Hospital Istituto di Ricovero e Cura a Carattere Scientifico (IRCCS), Florence, Italy; ^3^ Endocrinology Division, Section of Pediatrics, Meyer Children’s Hospital Istituto di Ricovero e Cura a Carattere Scientifico (IRCCS), Florence, Italy; ^4^ Department of Experimental and Clinical Medicine, University of Florence, Florence, Italy; ^5^ Immunoallergology Unit, Careggi University Hospital, Florence, Italy; ^6^ Flow Cytometry Diagnostic Center and Immunotherapy, Careggi University Hospital, Florence, Italy

**Keywords:** NFKB1, whole exome sequencing, common variable immunodeficiency, short stature, growth hormone

## Abstract

We report the case of a patient with common variable immunodeficiency (CVID) presenting with short stature and treated with recombinant human growth hormone (rhGH). Whole exome sequencing revealed a novel single-nucleotide duplication in the *NFKB1* gene (c.904dup, p.Ser302fs), leading to a frameshift and thus causing *NFKB1* haploinsufficiency. The variant was considered pathogenic and was later found in the patient’s mother, also affected by CVID. This is the first reported case of a patient with CVID due to *NFKB1* mutation presenting with short stature. We analyzed the interconnection between *NFKB1* and GH – IGF-1 pathways and we hypothesized a common ground for both CVID and short stature in our patient.

## Introduction

Short stature is defined by height more than 2 standard deviations (SD) below the mean for children of the same chronologic age, sex, and population group (1). Short stature and immunodeficiency often coexist in the wide spectrum of inborn errors of immunity (IEI). Indeed, the growth process may be affected by persistent inflammatory response due to recurrent and severe infections (2); moreover, several genetic syndromes may involve both the growth process and the immune system (3).

Common variable immunodeficiency (CVID) is a primary immunodeficiency (PID), characterized by primary hypogammaglobulinemia, and consequently increased infection susceptibility. Loss-of-function *NFKB1* variants are the most common monogenic cause of common variable immunodeficiency in Europe (4). NFKB1 belongs to the nuclear factor-kappa B (NFKB) family, a signal transduction multi-component pathway, involved in cell proliferation, cell survival, cell stress response, immune response, and inflammation. Mutations in several NFKB pathway genes may cause immunodeficiency (5, 6). NFKB signaling comprises a “canonical” pathway, mediated by NFKB1, and a “non-canonical” pathway, mediated by NFKB2.

After observing the clinical findings described in this case report, we reflected on the role that the NFKB pathway might play in linking immune dysregulation and growth disorders in children.

## Case description

In 2011, a 6-year-old boy was referred to our Immunology Department in Meyer Children’s Hospital because he had suffered from recurring episodes of pharyngotonsillitis and acute otitis media (AOM) since his preschool age, with no severe infections; also, his family history was suggestive of immunodeficiency. A few months earlier, the patient had been evaluated by the Endocrinology Department of our Hospital for short stature; of note, his mother also displayed short stature, with a height below 2 SD. The patient, born at term, was small for gestational age (SGA), with a birth weight of 2420 g (< -2 SD) and a length of 44 cm (< -3 SD). His postnatal course was unremarkable.

The family history of our patient was remarkable for humoral primary immunodeficiency. His maternal grandmother had suffered from recurrent episodes of gastroenteritis, one episode of salmonellosis, and two episodes of pneumonia; she had been treated with intravenous immunoglobulin replacement therapy for one year, before dying of meningoencephalitis at the age of 28. Furthermore, the mother of the patient had suffered from three episodes of pneumonia since the age of 30; her blood tests had revealed hypogammaglobulinemia with reduced serum levels of IgG (45 mg/dl), IgA (23 mg/dl), and IgM (16 mg/dl). Subsequently, the patient’s mother was diagnosed with common variable immunodeficiency (CVID), and she was qualified for immunoglobulin replacement therapy. During the course of her disease, she developed granulomatous hepatitis and refractory enteropathy.

After the initial visit to our Department, an initial workup of suspected immune deficiency was performed, showing decreased serum IgG (509 mg/dl; normal value for age 1164 ± 2SD) and IgA (37 mg/dl; normal value for age 113 ± 2SD) with normal IgM (72 mg/dl; normal value for age 121 ± 2SD), while analysis of lymphocyte subpopulations was normal; anti-tetanus and anti-HBs immunoglobulin titers were protective, while anti-diphtheria immunoglobulin titers were not protective.

Given the hypogammaglobulinemia and the family history of CVID, the patient was then followed up at our Immunology Department with biannual clinical and laboratory assessments. Due to recurring AOM, with adenoid hypertrophy and flat tympanogram at the ENT evaluation, in 2013 the patient underwent adenoidectomy, after which the recurring episodes of AOM abated. Over the years, the patient did not suffer from any severe infection and the patient and his family have always refused the start of any prophylactic or supportive treatment.

**Figure 1:**
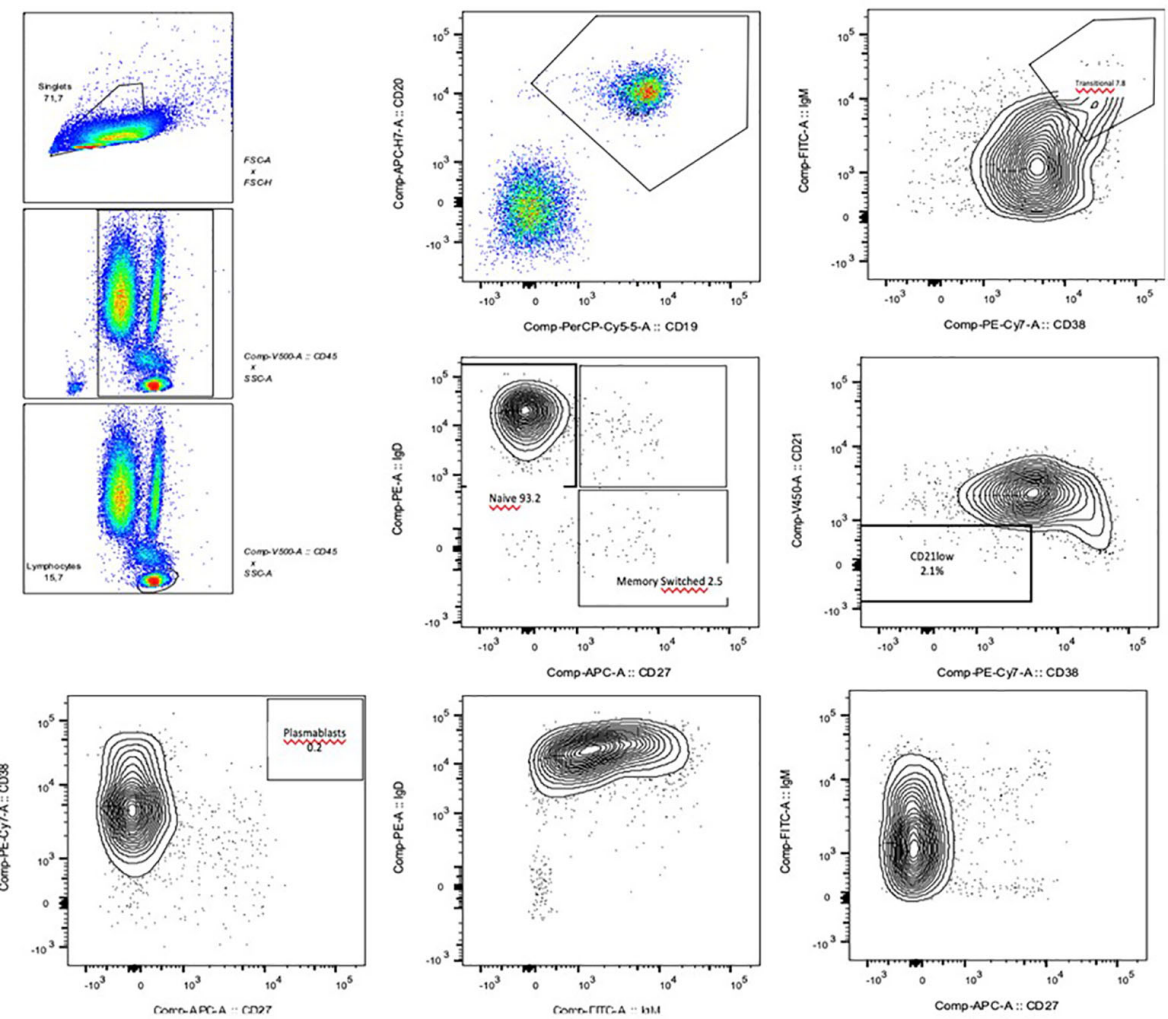
Flow cytometry images of the B cell phenotyping: the reduced levels of total and switched B memory cells and plasmablasts are clearly visible.

In 2016, comprehensive follow-up blood tests highlighted, in addition to persistent hypogammaglobulinemia (reduced IgG, 481 mg/dl, and IgA, 32.7 mg/dl, with normal IgM), abnormalities in lymphocyte subpopulations analysis (**Table 1**; **Figure 1**), in particular, a decrease in both the percentage and the absolute count of total memory B cells (30 per μl, 6.8% of B cells), with a prevalence of non-switched (CD27+IgD+) over switched (CD27+IgD-) memory B cells (4.3% *versus* 2.5% of B cells); anti-tetanus and anti-HBs immunoglobulin titers were protective, while anti-diphtheria immunoglobulin titers were not protective. The association of hypogammaglobulinemia and low switched B memory cells, in the absence of T cell immunodeficiency and of any other cause of immunodeficiency, allowed us to make the diagnosis of CVID in our patient, according to the European Society for Immunodeficiencies (ESID) criteria. Immune globulin replacement therapy was then recommended, but it was never administered due to family refusal.

**Table 1:** The results of the analysis of lymphocyte subpopulations of the patient in 2016, significant for reduced levels of total and switched memory B cells: this finding, in association with his preexisting hypogammaglobulinemia and his family history, pointed toward the diagnosis of CVID in our patient.

Variable	Reference Range	Patient
Total lymphocytes (per µI)	500–5000	1901
CD3+ (per µI)	570–2800	1217
CD3+CD4+ (per µI)	600–2000	836
CD3+CD8+ (per µI)	110–800	342
CD3+HLA-DR+ (per µI)	0–590	86
CD4+/CD8+ (per µI)	1–3.5	3.1
CD19+ (per µI)	100–500	437
CD3-CD16+CD56+ (per µI)	200–400	242
CD19+ (%)	6.4–22.6	23
Naive (lgD+CD27-) (%)	42.6–82.3	932
Memory non-switched (lgD+CD27+) (%)	7.4–32.5	4.3
Memory switched (lgD-CD27+) (%)	6.5–29.1	2.5
Transitional (lgM++CD38++) (%)	0.6–3.4	7.8
CD211ow (%)	0.9–7.6	2.1
Plasmablasts (CD27++CD38++) (%)	0.4–3.6	0.2
CD3+CD4+ (%)	28–64	48.4
CD4+ naive (CD45RA+CCR7+) (%)	16–100	64.2
CD4+ TCM (CD45RA-CCR7+) (%)	18–95	22
CD4+ TEM (CD45RA-CCR7-) (%)	1–23	10.8
CD4+ TEMRA (CD45RA+CCR7-) (%)	0–6.8	3
RTE (CD4+CD31 +) (%)	7–100	59
CD3+CD8+ (%)	12–40	15.6
CD8+ naive (CD45RA+CCR7+) (%)	6–100	70.1
CD8+ TCM (CD45RA-CCR7+) (%)	1–20	1.8
CD8+ TEM (CD45RA-CCR7-) (%)	14–98	17.5
CD8+ TEMRA (CD45RA+CCR7-) (%)	7–53	10.6
CD8+CD57+ (%)		4

**Figure 2:**
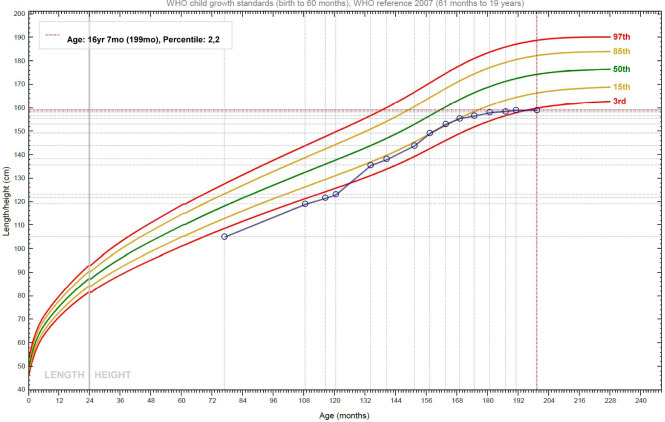
Growth curve of the patient: after the initial phase of catch-up growth, the height velocity declines, and the stature slowly approaches the target height.

Alongside this, the patient continued to be followed up by the Endocrinology Department. In 2015, when he was 10 years old, because of his short stature (< -2.5 SD) and reduced growth velocity (< 50° percentile), treatment with recombinant human Growth Hormone (rhGH) was started, in compliance with the regulations from the Italian Medicines Agency (AIFA) for rhGH treatment in children born SGA. The initial catch-up growth of the patient was good, with a height velocity of around 9 cm/year for the first two years of treatment, but this growth spurt coincided with his puberal development and decreased after four years of treatment (**Figure 2**). In accordance with national prescription guidelines, rhGH treatment was administered for 6 years, until discontinuation at the age of 16, after reaching the final adult stature. Overall, rhGH treatment was not plainly effective in our patient, with a final height below the third percentile.

Given the hereditary hypogammaglobulinemia and the short stature responsive to rhGH therapy, we proceeded to perform a genetic analysis. We included the patient and his mother in the “CVIDOME” program: the project, funded by the Tuscany region, aimed at investigating the genetic causes of CVID through whole-exome sequencing.

In our patient, the genetic analysis revealed a novel heterozygous single-nucleotide duplication (NM_003998.4) in the *NFKB1* gene, c.904dup (p.Ser302fs), leading to a frameshift. Equivalent duplications in the *NFKB1* gene leading to the same frameshift were reported in several other patients with CVID (4, 7, 8). The variant, later found also in the mother, was considered responsible for the clinical picture and thus pathogenic.

The patient is still followed-up at our center; he has not suffered from any infectious events or autoimmune phenomena. At the present time, no specific therapy for the disease is possible in our patient. Nonetheless, the identification of the genetic defect underlying his condition might allow, in the future, to start a potential disease-modifying treatment, such as targeted therapy, if ever discovered, or hematopoietic stem cell transplantation (HSCT), if clinically indicated.

## Discussion

We report for the first time the case of a patient with hypo-gammaglobulinemia and short stature due to a pathogenic variant in the *NFKB1* gene.

The nuclear factor-kappa B (NFKB) family is a complex multi-component pathway that is critical for immune regulation. Five transcription factors have been identified in this pathway: NFKB1 (p105/p50), NFKB2 (p102/p52), RelA/p65, RelB, and c-Rel. These proteins share a Rel homology domain (RHD) implicated in homo- and heterodimers formation which drive or inhibit target gene expression by DNA binding in the nucleus. *NFKB1* encodes for the transcription factor NFKB1, which is implicated in cell proliferation and survival, inflammation, and immune response (5, 7). Therefore, NFKB aberrant signaling may be implicated in the pathogenesis of a large spectrum of diseases ranging from neoplasia (9) to autoimmune disorders or immunodeficiency (5, 10–12).

As far as immunodeficiency is concerned, *NFKB1* loss-of-function variants are the most common monoallelic cause of CVID in the European population (4), accounting for 4% of CVID cases in the analyzed cohort. Among the other CVID patients, those with *NFKB1* mutations displayed a higher prevalence of autoimmunity and malignancy, known negative prognostic factors.

The phenotype of NFKB1 haploinsufficiency encompasses a wide range of manifestations, including combined B cell and T cell dysfunction, EBV-driven lymphoproliferation (9), autoimmunity, and autoinflammatory disorders like Behçet disease (13). Affected patients typically present a reduced number of switched memory B cells and an increased number of non-switched memory B cells (7, 14). Given the relevant frequency of noninfectious manifestations, NFKB1 haploinsufficiency should be considered an immune dysregulation disease, not only a primary immunodeficiency.

Growth hormone (GH) deficiency and insensitivity have not been reported among the numerous clinical features in the wide spectrum of *NFKB1* haploinsufficiency; the role of *NFKB1* mutations in growth processes has not been widely studied. A notable exception is a paper published in 2013 (15), reporting the result of a prospective, cross-sectional, epidemiogenetic study conducted between 2008 and 2010 (The EPIGROW study) in order to identify biological and genetic characteristics of children with idiopathic short stature (ISS) and SGA in a large European cohort. Laboratory investigations showed that 53% of patients with ISS had an insulin-like growth factor 1 (IGF-1) deficiency. Whole exome sequencing was then performed in these children and in ethnicity-matched controls. The genetic analysis of EPIGROW highlighted the possible connection with the short stature of pathways not directly related to the GH - IGF-1 axis. Indeed, genes involved in transcriptional regulation and in growth factor signaling (*NFKB1* and *ZBTB38*) resulted to have a statistically significant association with short stature. *NFKB1* variant was twice as frequent in cases compared with controls in this study.

Of note, short stature is often seen in the context of primary immunodeficiency syndromes: this finding has been classically described as multifactorial, primarily due to the chronic inflammatory process arising from recurrent infections. Nevertheless, while immune compromise may certainly contribute to determining short stature in some cases, our patient did not experience severe infections that could explain his growth retardation; thus, a primary dysfunction of the GH - IGF-1 could be hypothesized to underlie his short stature.

It is worth noting that an association between immunodeficiency and distinct endocrinopathies has been described in several primary immunodeficiencies, the two most well-known being IPEX and APECED, but neither of them usually presents with GH deficiency/insensitivity.

Recently, a syndrome encompassing multiple pituitary abnormalities along with an immune phenotype of CVID has been described and named DAVID (Deficient Anterior pituitary with Variable Immune Deficiency) syndrome. The first reported patients were screenees from a French cohort of ACTH deficiency cases without a genetic diagnosis, who were also found to have CVID: the authors did not deem this association to be casual and proposed the name “DAVID association” (16). Of note, the study investigated whether ACTH deficiency was the result of a common genetic lesion or the consequence of autoimmune hypophysitis (in the context of the autoimmune manifestations of CVID). The patients did not display any autoimmune manifestation, and no anti-pituitary autoantibodies were detected in their serum; thus, even though the authors did not find pathogenic variants in the tested genes (*LIF*, *IKAROS*, and *EOS*), they postulated a genetic basis for DAVID syndrome. In 2013, the genetic defect causing DAVID syndrome was identified in pathogenic variants of the *NFKB2* gene, particularly in the C-terminal region, leading to the disruption of p100 phosphorylation, inhibition of processing into the p52 active form, and prevention of nuclear translocation (17). Later, other anterior (18) and posterior (19) pituitary abnormalities were reported in association with CVID (20) in patients with *NFKB2* pathogenic variants; interestingly, the second most common alteration, after ACTH deficiency, was GH deficiency.

The pathogenetic mechanism of this association has not been clearly defined. *NFKB2* is known to be involved in thymic *AIRE* expression, supporting the possibility that an autoimmune phenomenon is the cause of endocrinological anomalies in DAVID syndrome; nonetheless, anti-pituitary autoantibodies have never been detected in the blood of patients with this syndrome so far. Vice versa, the hypothesis of a common genetic basis of both immune system dysfunction and pituitary abnormal development is not supported by a recent *in vivo* study (21): the role of *NFKB2* expression in pituitary organogenesis was apparently excluded since no anatomical or histological difference was found between the pituitaries of wild-type and mutated (in the ortholog gene *Lym1*) murine models.

Despite the absence of any reported case of CVID and anterior pituitary dysfunction related to NFKB1, it is worth remembering that the two pathways, “canonical” and “non-canonical” are interconnected: in addition to sharing many target genes, NFKB2 p100 precursor protein acts as an inhibitor of NFKB1 through binding it with an ankyrin repeat domain (ARD), similar to IκB (22). Furthermore, NFKB1 expression has been detected in adult mouse pituitary cells nearly at the same level as NFKB2 (21).

Janssen et al. (23) described the case of a patient with hyper immunoglobulin M–like immunodeficiency syndrome, ectodermal dysplasia, growth retardation, and GH resistance: the genetic analysis revealed a mutation in the *NFKBIA* gene, coding for IκBα, a key component of the “canonical” NFKB pathway. In a subsequent study, Wu S. et al. (24) cultured the fibroblasts of the patient, obtained from a skin biopsy, and failed to find any effect of GH and IGF-1 stimulation on cell proliferation and *TDAG51* (a target gene for IGF-1) expression. This finding underlined the role of an intact NFKB signaling pathway for the growth-promoting effects of the GH - IGF-1 axis, as previously suggested by murine chondrocytes models (25). Of note, IκBα has no role in the “non-canonical” NFKB pathway: thus, the disruption of the downstream signaling of IGF-1 must have been entirely due to the alteration in the “canonical” NFKB pathway (i.e., NFKB1 mediated).

**Figure 3:**
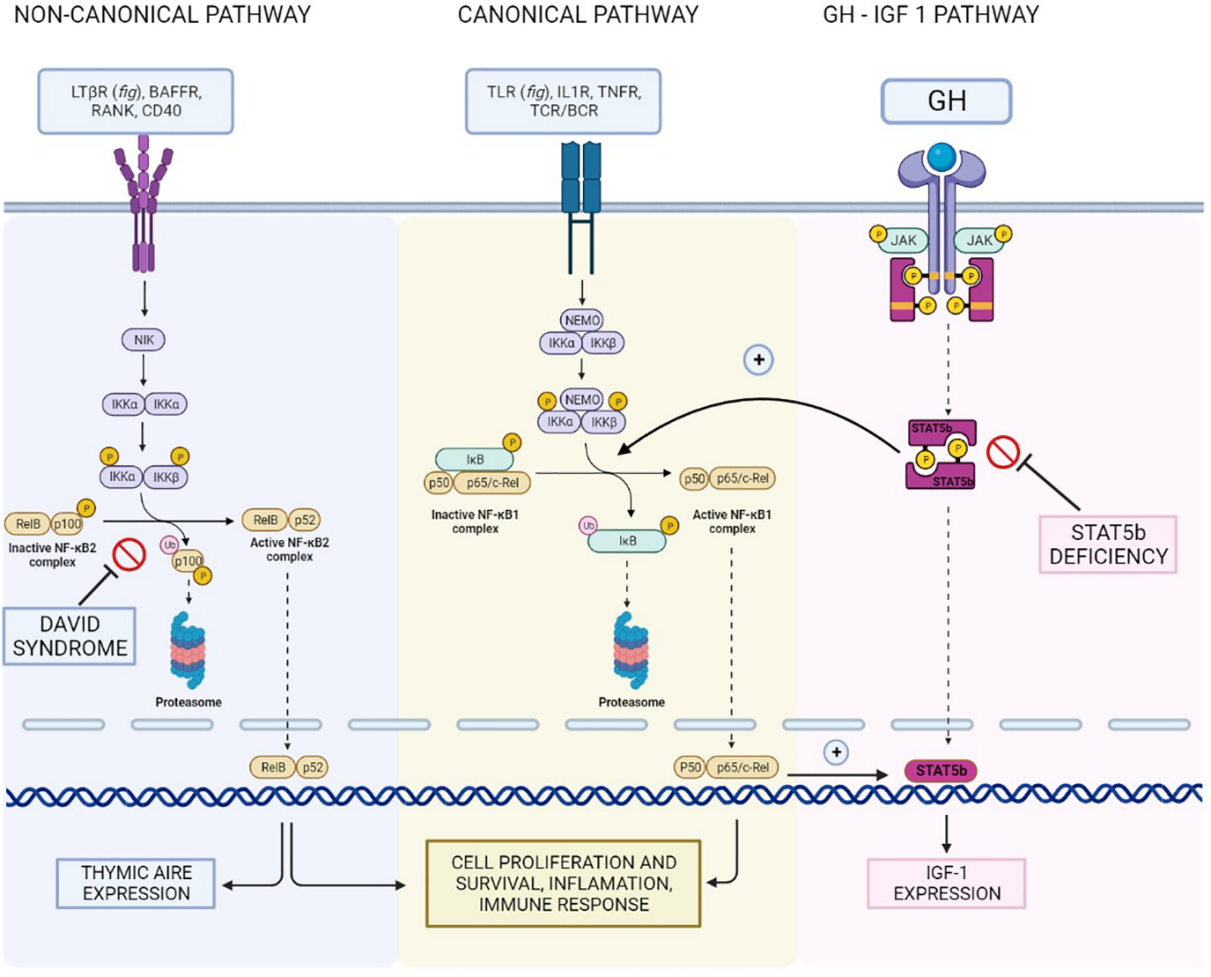
Reconstruction of the NFKB1/NFKB2 and GH - IGF-I pathways and their interconnections, illustrating the hypothetical shared basis for the different clinical manifestations of our patient.

Furthermore, the study took a deeper look at the crosstalk between NFKB and STAT5b pathways: it is known that STAT5 signaling induces nuclear translocation of NFKB, which then acts as a transcription factor for many genes including *STAT5*. The JAK-STAT pathway is a key component of the GH signaling axis, promoting transcription of the *IGF1* gene (26, 27). STAT5b deficiency classically presents with GH insensitivity and immunodeficiency (28–30): thus, it appears reasonable to suppose that a similar phenotype may arise from alterations in the NFKB pathway, given its connections with the STAT5 pathway (**Figure 3**). Nonetheless, our patient did not display signs of GH resistance, even though his response to rhGH therapy was suboptimal; this finding underscores that these two pathways, despite their interactions, have distinct roles in GH signaling. Besides, it is worth remembering that NFKB1 haploinsufficiency and STAT5b deficiency cause distinct immunodeficiency phenotypes (31, 32).

In conclusion, even if strong evidence supporting a shared pathogenetic basis for this association is lacking, it seems reasonable to hypothesize that both CVID and short stature in our patient resulted from a disruption in the complex interplay between the various components of the NFKB pathway and in the crosstalk with other essential signaling pathways. In particular, given the role of NFKB2 in a syndrome encompassing CVID and endocrinopathies, the hypothesis that *NFKB1* pathogenic variants might underlie GH deficiency in the context of CVID seems at least grounded on biological plausibility.

## Conclusions

- This is the first case report which describes the role of a novel *NFKB1* mutation affecting not only the immune system but also the growth process. We identified a novel single-nucleotide duplication in the *NFKB1* gene leading to a frameshift classified as pathogenic: the phenotype of the affected pediatric patient showed the coexistence of both humoral immunodeficiency and GH deficiency.
- Growth disorder is a common clinical problem in children therefore, given the frequent association between short stature and immunodeficiency, in children who are falling off their growth curves, the pediatrician should also consider defects in the immune system in the differential diagnosis and should then be alert about the warning signs of IEI.

## Data availability statement

The raw data supporting the conclusions of this article will be made available by the authors, without undue reservation.

## Ethics statement

Written informed consent was obtained from the individual(s), and minor(s)’ legal guardian/next of kin, for the publication of any potentially identifiable images or data included in this article.

## Author contributions

SR: Conceptualization, Data curation, Investigation, Project administration, Resources, Supervision, Visualization, Writing—original draft, and Writing—review & editing. SA-R, BP, and NC: Data curation, Formal Analysis, figures creation, Visualization, Writing—original draft, and Writing—review & editing. AV, AM, CC, FB, LL, and SS: Data curation, Validation, and review & editing. CA, FA, and LM: Conceptualization, Project administration, Supervision, Validation, review & editing, and Funding acquisition. All authors contributed to the article and approved the submitted version.
